# Microenvironment derived from metanephros transplantation inhibits the progression of acute kidney injury in glycerol-induced rat models

**DOI:** 10.1080/0886022X.2019.1708393

**Published:** 2020-01-03

**Authors:** Kailin Li, Yuan Chen, Jianye Zhang, Yong Guan, Chao Sun, Xian Li, Xiaoshuai Xie, Denglu Zhang, Xin Yu, Tongyan Liu, Xufeng Zhang, Feng Kong, Shengtian Zhao

**Affiliations:** a Department of Central Research Lab, The Second Hospital of Shandong University, Jinan, China; b Key Laboratory for Kidney Regeneration of Shandong Province, Jinan, China; c Department of Urology, The Second Hospital of Shandong University, Jinan, China; d Shandong Provincial Hospital, Jinan, China; e The Second Hospital of Shandong University, Jinan, China; f The Affiliated Hospital of Shandong University of Traditional Chinese Medicine, Jinan, China; g Karolinska Institutet Collaborative Laboratory for Stem Cell Ressearch, Shandong University, Jinan, China

**Keywords:** Metanephros transplantation, acute kidney injury, metanephros microenvironment, nephrogenic repair

## Abstract

**Background:**

Embryonic metanephros is the mammalian renal anlagen, which is considered as a potential source for the regeneration of functional whole kidneys. Some studies reported that metanephros implanted into unilateral nephrectomized animals can develop into kidney tissue. However, kidneys are nephrotoxic in renal failure patients, and whether metanephros can grow in nephrotoxic has not been reported. This study aims to investigate the growth of metanephros in acute nephrotoxic environment and analyze the therapeutic effect of metanephros microenvironment on acute kidney injury (AKI).

**Methods:**

AKI was induced in 200 g Wistar rats by giving intramuscular injections of 50% glycerol (10 mL/kg) in their hind limbs. 45 rats were divided randomly into three groups (control, glycerin, and metanephros). Metanephros group was transplanted two metanephroi (embryonic day 15) into the renal capsule of AKI rats. Glycerin group was AKI rats without transplantation. Control group was untreated.

**Results:**

Mature glomeruli and tubules were detected in the grafts in metanephros group, which means that metanephroi can grow into tissues with mature kidney structure under acute nephrotoxic. Then, we assessed the renal function of host rats and found that there were fewer tubular necrosis in metanephros group than glycerin group, and the serum creatinine and urea nitrogen were significantly lower in metanephros group than glycerin group.

**Conclusion:**

These results suggested that embryonic metanephroi can grow into tissues with mature kidney structure under acute nephrotoxic, and the graft microenvironment was effective in inhibiting the progression of AKI, which provides a new approach for the treatment of acute renal injury.

## Introduction

The complexity of the anatomical structure of the kidney makes it one of the most difficult challenges in the field of organ regeneration [1]. The embryonic metanephros is the mammalian renal anlagen, which is thought to develop into all cell types of adult kidney, and is considered as one of the potential sources for the regeneration of functional whole kidneys [2,3]. The metanephros on the 15th day of embryonic development (E15) is composed mainly of undifferentiated metanephric mesenchyme with rudimentary epithelial structures [4], which has been used in many studies of kidney regeneration [3,5,6]. Metanephros has been suggested as less immunogenic and more feasible for transplantation because of the lack of antigen-presenting cells that recognize allogeneic or xenogeneic antigens in the early stages of the embryo [7]; donor antigens, such as major histocompatibility complex class I and II, may not be expressed by developing organs [8]. So transplantation of the embryonic metanephros could partially avoid the immuno-rejection problem. Our recent research showed that metanephroi transplanted into unilateral nephrectomy rats can differentiate into a tissue microenvironment with vascularized glomeruli and proximal tubules [9]. The blood vessels of the grafts were originated from the host [6,10]. As we all known, the kidneys of patients with renal failure were injury or nephrotoxic, whether embryonic metanephros can grow and differentiate in the environment of kidney injury determines the application prospects of embryonic metanephros transplantation in the treatment of nephropathy.

It is reported that the microenvironment derived from transplanted metanephros can induce the differentiation of exogenous stem cells into renal cells [11]. The microenvironment derived from transplanted metanephros could maintain a blood pressure of the host animal and inhibit the progression of vascular calcification in rats with adenine-induced renal failure by producing renal hormones, including EPO and renin [12,13]. These results suggested that the microenvironment might be useful in the treatment of nephropathy; however, there have been no reports that whether the metanephros microenvironment is beneficial in the treatment of AKI.

AKI is a high incidence and high mortality clinical syndrome characterized by a sudden decline of renal function. The etiology of AKI is complicated, such as rhabdomyolysis, hemorrhagic shock, infection, urethral obstruction, and so on [14,15]. Epidemiological investigation showed that the incidence rate of AKI patients in ICU was 30–50% and the mortality rate was 63% [16]. It takes between 6 months and a year for the survival patients to recover their renal function completely. About 19–31% of the patients with AKI develop chronic nephropathy or end-stage renal failure due to poor renal repair [17,18]. Therefore, early therapeutic interventions to improve the nephrotoxic environment plays a key role in the treatment of AKI, which can effectively prevent its progression to chronic nephropathy [19–22]. Recent studies have used glycerol-induced acute kidney injury rats as a useful model of AKI [23,24].

In the present study, embryonic metanephroi were implanted into rats with acute nephrotoxicity, and then the differentiation of the grafts and the therapeutic effect of the metanephroi microenvironment on acute kidney injury were detected. The results suggest that embryonic metanephroi can grow into tissues with mature kidney structure under acute nephrotoxic, and the metanephroi microenvironment contributes to the suppression of the progression of AKI.

## Materials and methods

### Animals

Animals were provided by Shandong University Laboratory Animal Center, Jinan, China. Forty-five male Wistar rats weighing 190–210 g were housed in the Animal Center of the Second Hospital of Shandong University under a controlled environment at a controlled temperature and humidity with a 12-h light/dark cycle. The experimental protocol was approved by the Animal Ethics Committee of the Second Hospital of Shandong University (KYLL-2018(KJ)A-0039).

### Experimental design

Rats were divided randomly into three groups. The control group (*n* = 15) were given intramuscular injections of physiological saline in their hind limbs. The glycerin groups (*n* = 15) and the metanephros groups (*n* = 15) were given intramuscular injections of 50% glycerol (10 mL/kg) in their hind limbs. Blood creatinine and urea nitrogen were detected by orbital blood sampling 24 h after intramuscular injection to verify the success of acute kidney injury (AKI) model. Rats in the metanephros groups underwent a metanephros transplant 24 h after intramuscular glycerin injection, with two metanephroi at a time. Then, the rats were weighed at 1w, 2w, and 3w after transplantation, and the kidneys and blood were collected for renal gravity, renal volume, creatinine, urea nitrogen, with five rats at each time point.

### Metanephros procurement and transplantation

The 15-day pregnant rats were anesthetized with pentobarbital, the embryo was taken out by opening abdominal cavity under aseptic condition, and the fetal kidney was dissected under a stereomicroscope (Leica, Wetzlar, Germany) and placed in sterile PBS. The recipient rats fasted one day in advance. After pentobarbital anesthesia, we opened the left kidney side of the back. After exposure of the kidney, the renal capsule was cut into a small mouth, and the two metanephroi were placed through the opening at the upper and lower poles of the receptor kidney, respectively. The rats were euthanized for analysis at predefined time points.

### Determination of Scr and BUN

The blood of rats was collected through the orbit, and the serum was obtained by centrifuging at 3000 rpm for 10 min. The Serum creatinine (Scr) and blood urea nitrogen (BUN) levels of all analytes were measured using an autoanalyzer (COBAS 8000, Roche Diagnostics, Basel, Switzerland).

### HE staining and immunofluorescence

The renal tissue was fixed in 4% PFA and made into paraffin sections with a thickness of 4 μm. According to the specification of HE staining kit, after dewaxing, rehydration, hematoxylin staining for 10 min, eosin for 2 min after differentiation, then, dehydration, wax immersion, and sealing tablets were performed.

Indicators tested by immunofluorescence include WT1, PAX2, CD31. All operations were based on the manufacturer’s protocols.

### Statistical analysis

All data were statistically analyzed by SPSS16.0, and *T*-test was used between samples. *p* > 0.05 means no statistical difference. 0.05>*p* > 0.01 means there is a statistical difference. *p* < 0.01 means the statistical difference is extremely significant. The measured data were expressed as the mean ± standard deviation, and the column diagram was made with GraphPad Software.

## Results

### AKI induced by intramuscular injection of glycerol

**Figure 1.**
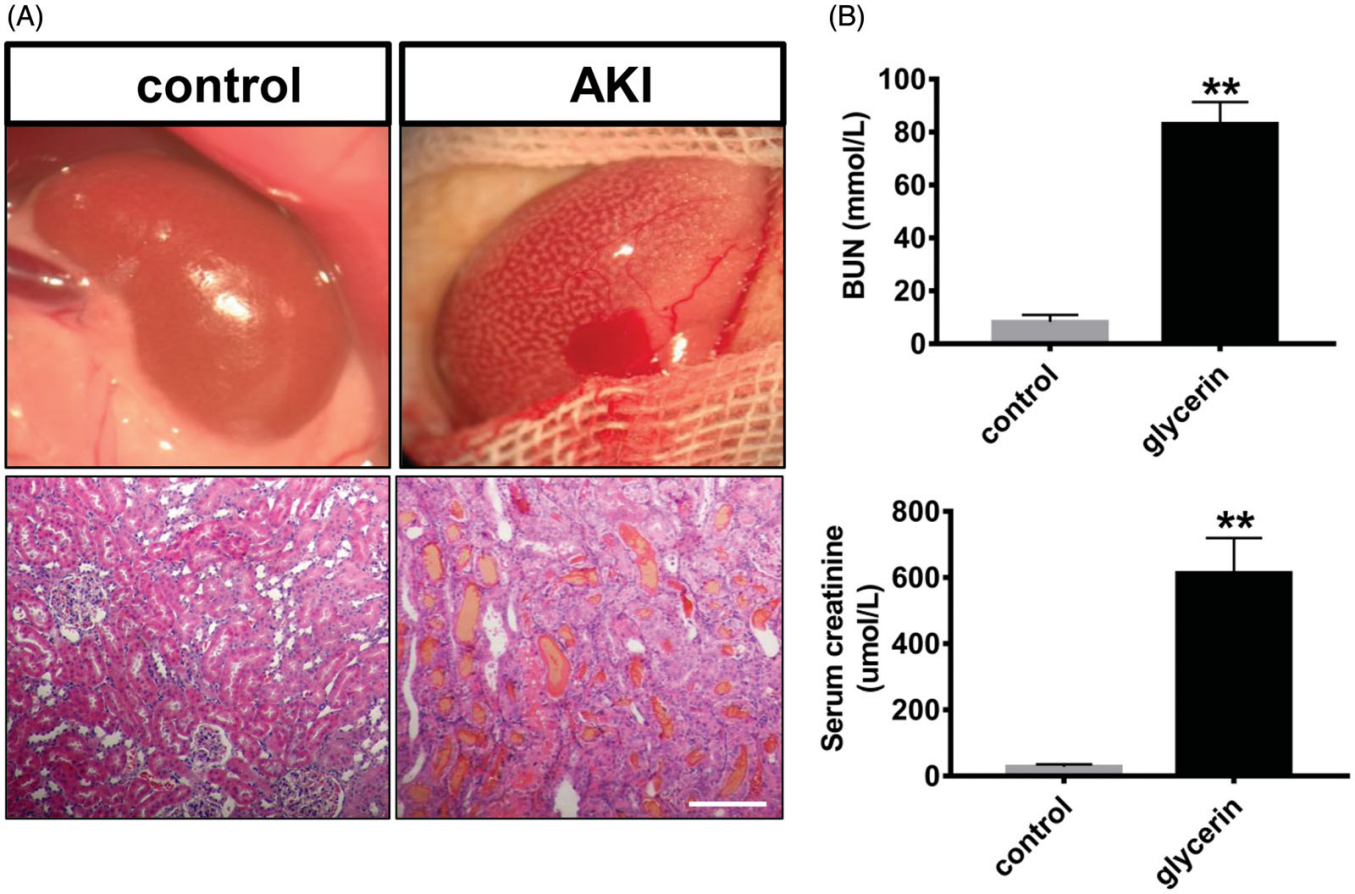
AKI rats model were induced by intramuscular injection of glycerol. Twenty-four hours after glycerol injection, the release of a large amount of myoglobin resulted in acute kidney injury, and the kidney was enlarged and textured (A). The levels of serum creatinine and urea nitrogen in the AKI group were significantly higher than the normal control group (B). Bar: 100 μm.

The morphologic and histopathological analysis of kidney tissue was performed after glycerol administration. As shown in Figure 1(A), after 24 h of glycerol injection, the kidney was enlarged and textured macroscopically. Pathological examination revealed that a large number of myoglobin cast and atrophy renal tubules in the AKI kidney.

Then, we evaluated renal insufficiency and renal damage by analyzing the levels of the serum creatinine and urea nitrogen. The results showed that the levels of serum creatinine and urea nitrogen in AKI group were significantly higher than the control group (Figure 1(B)), which indicate that the renal function of the AKI group decreased sharply, and the animal model was made successfully.

### Generation of Neo-kidney from metanephros transplantation in the acute nephrotoxic environment

**Figure 2.**
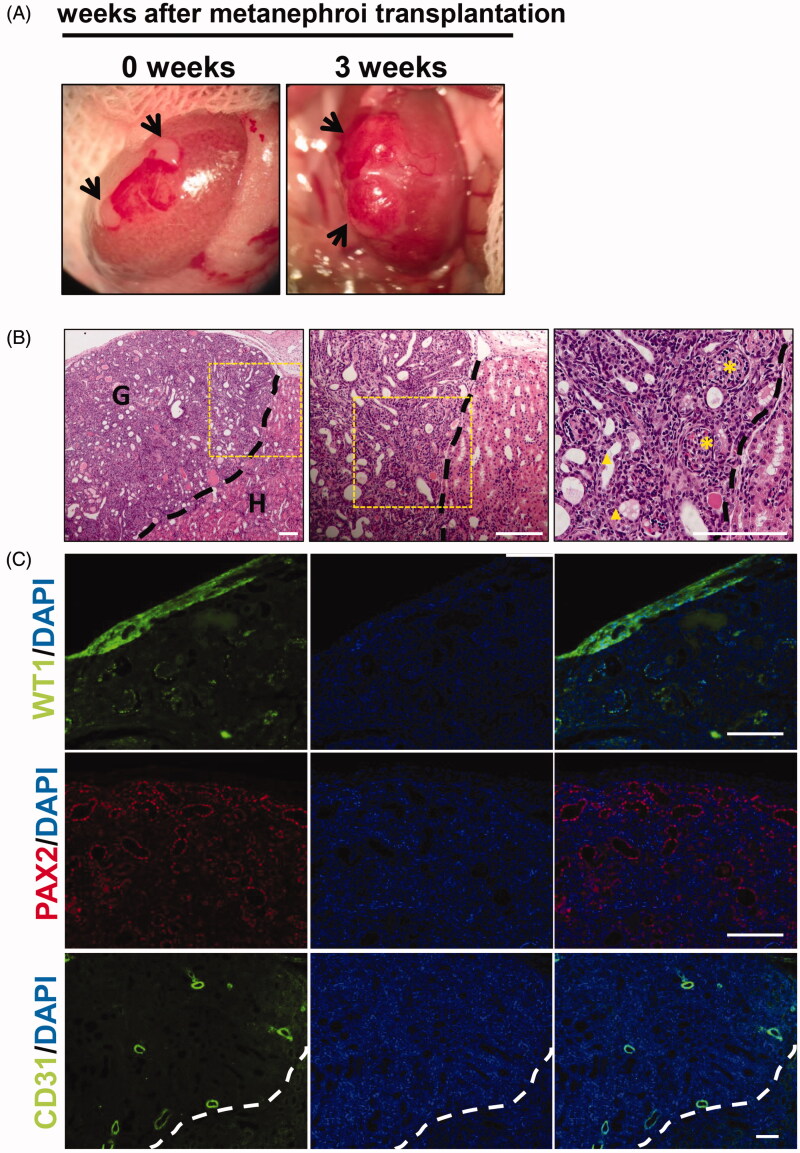
*In vivo* development of embryonic metanephros in acute nephrotoxic environment. (A) The process of transplanting metanephroi under the capsule, two metanephroi were transplanted to the renal capsule of AKI rats, and 3 weeks later, the grafts were found to be significantly larger and vascularized. (B) HE staining showed that mature glomeruli and tubules were detected in the grafts, the left side of the black dotted line is the graft and the right side is the host. (C) Histologic analysis revealed that WT-1-positive podocytes, PAX2-positive renal tubular cells, and CD31-positive vascular endothelial cells were detected in the grafts. G: graft; H: host; Bars: 100 μm.

As shown in Figure 2(A), two metanephroi were transplanted to the renal subcapsule of AKI rats. Three weeks later, the kidney rudiment grew up to be significantly larger and vascularized. Developed kidneys that were subjected to immunofluorescence and paraffin sections were observed by light microscopy. HE staining showed that mature glomeruli and tubules were detected in the grafts. The left side of the black dotted line is the graft and the right side is the donor kidney (Figure 2(B)). Immunofluorescence analysis revealed that WT-1-positive podocytes, PAX2-positive renal tubular cells, and CD31-positive vascular endothelial cells were detected in the grafts (Figure 2(C)), suggesting that developed metanephroi were well differentiated in acute nephrotoxic.

### Metanephroi transplantation alleviates glycerol-induced renal dysfunction and damage

**Figure 3.**
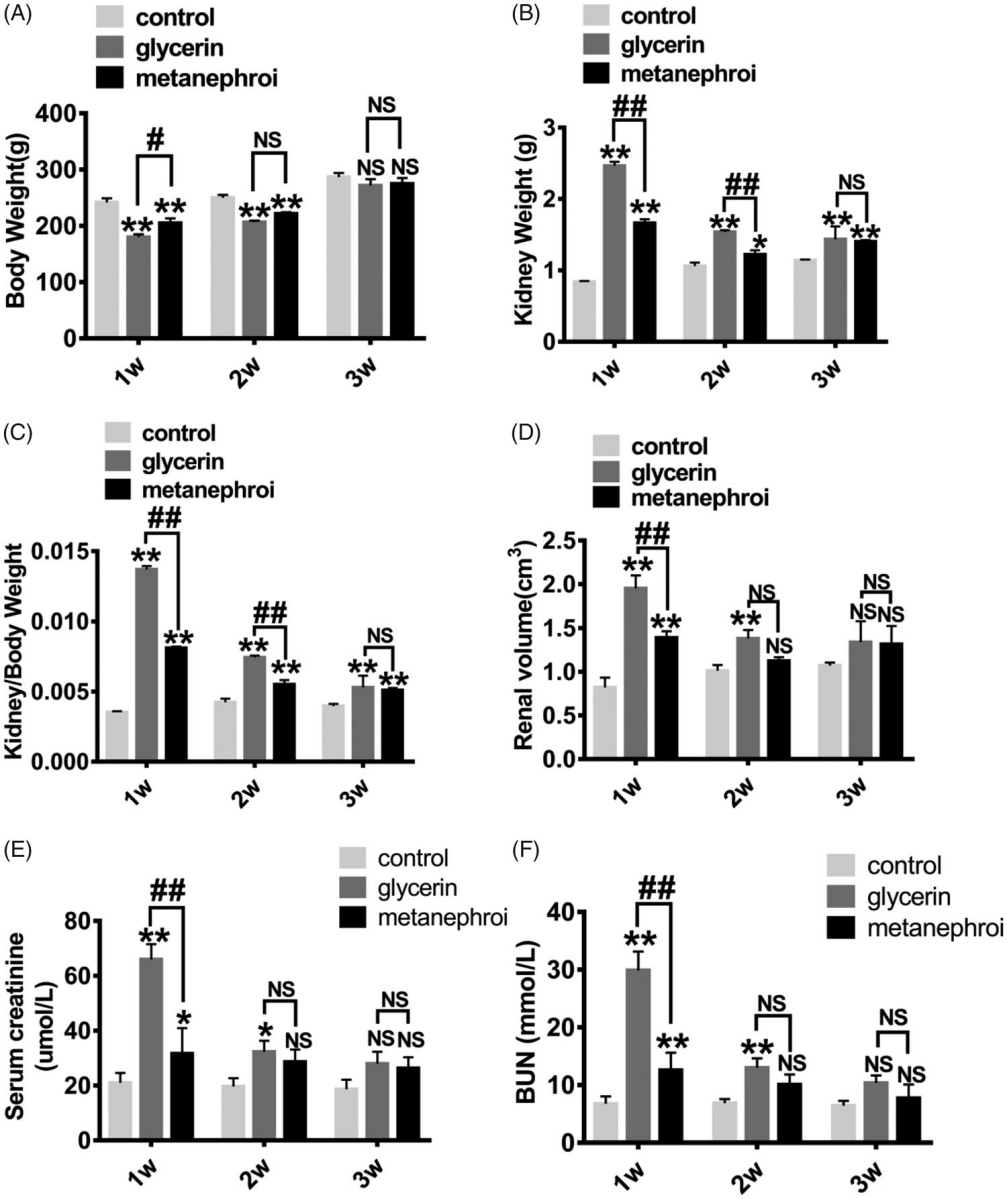
Representative changes in kidney at different time points after metanephroi transplantion. (A) The glycerol group and the metanephroi group showed significant weight loss, while the glycerol group showed more significant weight loss than the metanephroi group at the first and second weeks. There was no significant difference among the three groups at the third week. (B) The weight, (C) Kidney weight to body weight ratio and (D) volume of the kidney in the glycerol group increased significantly at 1 and 2 weeks, which was higher than the metanephroi group. The effect of metanephroi on glycerol induced changes in (E) Scr and (F) BUN. After metanephroi transplantation, the Scr and BUN levels decreased significantly at all time points compared with the glycerol group. *p < 0.05, **p < 0.01 for the glycerol group and the metanephroi group compared with the control group. ^#^*p* < 0.05, ^##^*p* < 0.01 for the glycerol group compared with the metanephroi group.

To investigate the therapeutic effect of the metanephros microenvironment on AKI, changes of body weight, renal weight, renal volume were analyzed among the three groups. Figure 3(A) shows that both the glycerol group and the metanephroi group showed significant weight loss compared with the control group, while the glycerol group lost more weight than the metanephroi group at the first and second weeks after metanephroi transplantation. There was no significant difference among the three groups at the third week. In contrast, the kidney weight and kidney/body weight ratio in the glycerol group and metanephroi group increased significantly at the first and second weeks, and the former increased more (Figure 3(B,C)). The changes in the volume of kidney followed the same trend in the glycerol and metanephroi groups (Figure 3(D)).

As two principal clusters of renal function biomarkers, the serum creatinine(Scr) and blood urea nitrogen(BUN) were recorded in this study. The Scr and BUN levels were significantly elevated at 1 week and were gradually decreased at 2 and 3 weeks in glycerol group. Interestingly, after metanephroi transplantation, the Scr and BUN levels decreased significantly at all time points compared with the glycerol group (Figure 3(E,F)).

These findings suggested that the metanephros microenvironment inhibit the progression of AKI and played a significant role in maintaining body weight, renal weight, renal volume, and the levels of Scr and BUN in acute nephrotoxicity.

### Metanephros microenvironment improves the kidney damages in histology and decreases KIM-1 expression after glycerol-induced AKI

**Figure 4.**
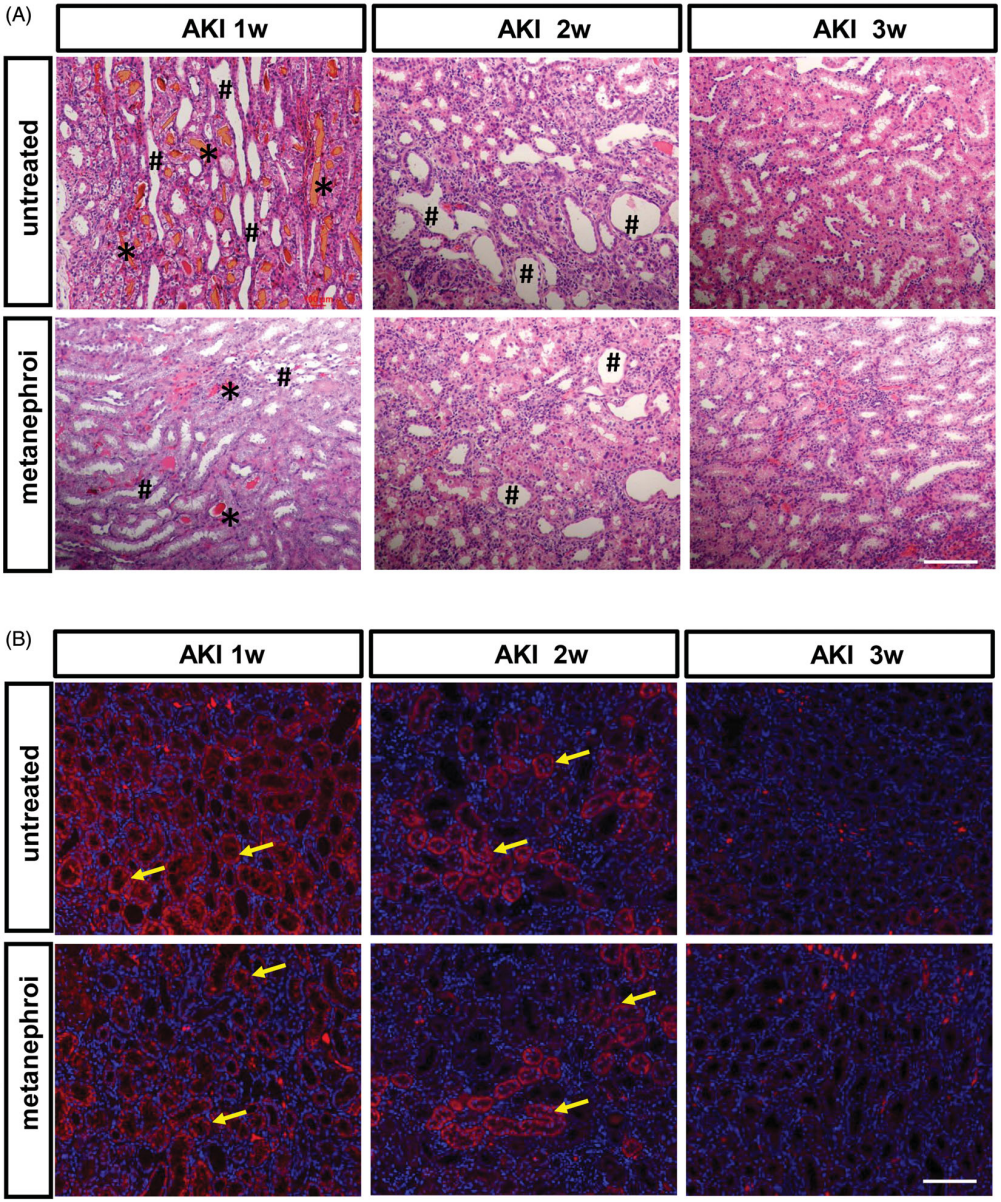
Effect of metanephroi microenvironment on renal injury in rats subjected to glycerol-induced AKI. (A) Representative morphological changes in the kidney assessed by H&E staining at different time points between the glycerol and the metanephroi groups. *, myoglobin cast. **^#^**, denotes necrotic tubules (B) KIM-1 levels at different time points between the glycerol and the metanephroi groups were analyzed by immunofluorescence. KIM-1-positive staining (Red fluorescence) was observed on the proximal tubular epithelial cells and damaged tubules (arrows). Bars: 100 μm.

Associated with the renal dysfunction, glycerol group rats showed widespread damage both in cortex and medullar region of the kidney. Extensive proximal tubular necrosis throughout the corticomedullary region, tubular dilatation and vacuolation, tubular necrotic lysis, cellular micro debris into the tubular lumen and a large number of atrophic renal tubules and myoglobin tubules were detected at 1 week, and gradually decreased at 2 and 3 weeks. Metanephros transplantation can significantly improve the histopathological changes of kidney induced by glycerol. The degree of tissue damage was less in the metanephros group compared with the glycerol group, as indicated by smaller areas of tubular epithelial necrosis and vacuolation (Figure 4(A)).

KIM-1 is a sensitive biomarker of kidney injury, and was used to evaluate kidney damage and repair after AKI. The expression of KIM-1 was analyzed by immunofluorescence. As shown in Figure 4(B), the expression of KIM-1 was very high at the first week after glycerol injection and decreased gradually in the second and third weeks. The changes in the expression of KIM-1 followed the same trend in the metanephros group. However, rats in metanephros group exhibited less KIM-1 staining distributed in tubular epithelial cells, and there were fewer KIM-1-positive tubules compared with glycerol-treated rat kidneys at the first and second weeks.

## Discussion

Therapeutic kidney regeneration has been the focus of many researchers as an ultimate therapy. The embryonic metanephros, with less immunogenic, is a promising potential source for the generation of a functional whole kidney [25]. However, it has been reported that renal proximal tubular cell regeneration is inhibited in a nephrotoxic environment [12,13,26–28]. Consequently, whether embryonic metanephros can grow and differentiate in nephrotoxic environment determines its application prospects in the treatment of nephropathy. In this study, we first demonstrated that the metanephroi could grow in AKI rats. Abundant blood vessels, mature glomeruli, and renal tubules ensured that the developed metanephroi might function well. What's more, we found that metanephroi microenvironment could effectively shorten the recovery time of AKI compared with the control rats. The body weight, kidney weight, kidney volume, serum creatinine (Scr), and blood urea nitrogen (BUN) were recovered faster in the metanephroi transplant rats than the untreated. KIM-1, a biomarker of kidney injury that is localized to damaged epithelial cells in the renal proximal tubule, was continuously expressed during the processes of kidney injury and recovery after AKI [29]. We found that rats in metanephros group exhibited less KIM-1 expression than the glycerol group at all time points, suggesting that the metanephros microenvironment was much more effective in decreasing KIM-1 levels. In addition, we are concerned that the metanephros microenvironment is not effective enough at the third week, which may be due to the regenerative repair ability of renal tubular epithelial cells in AKI [30]. In this study, we mainly focus on the therapeutic effect of the metanephros microenvironment on the early stage of AKI.

Early treatment of acute renal injury is very important to prevent the deterioration of acute renal injury [31]. Early intervention can effectively promote the recovery of acute renal injury and avoid the outcome of chronic renal injury [32,33]. In previous studies, it has been reported that AKI is treated by drugs, such as suramin injected intravenously, quinacrine injected intraperitoneally, and so on [24,32,34,35]. In this study, we selected a 15-day embryo kidney and transplanted it under the renal capsule of the recipient rats. Compared with the traditional methods, such as intravenous injection and intraperitoneal injection [36,37], subcapsular metanephroi transplantation can ensure the continuity of treatment of recipient kidney. Moreover, because the metanephroi microenvironment grows under the renal capsule, its protective effect on the kidney is more targeted. Previous studies have determined that metanephroi microenvironment plays an important role in renal regeneration [10,38,39], prevention of vascular calcification [12], maintenance of blood pressure [13], and so on.

However, there are some limitation in this study, the metanephros cannot survive for a long time *in vivo*, urine production will lead to dilated ureters, resulting in hydronephrosis, which will lead to renal fibrosis after 4 weeks [3,6], and ethical problems will arise when it is applied to human beings. In addition, the mechanism of protective effect of metanephroi microenvironment on AKI remains unclear. Firstly, we hypothesize that the inhibition of AKI by metanephroi microenvironment may be related to the characteristics of stem cells retained [11]. Some types of acute renal failure are reportedly treatable by transplantation of tissue- or bone-marrow–derived renal stem cells [40,41]. Secondly, inflammatory response and oxidative stress are associated with AKI [24,35]. We will explore the function of metanephroi microenvironment in nephrotoxic environment whether it can downregulate the inflammatory response and oxidative stress in AKI.

In conclusion, we have demonstrated that the metanephros could grow into tissues with mature kidney structure in the acute nephrotoxic environment, which provides a research basis for the application of embryonic kidney-derived regenerated kidney in the treatment of nephropathy. Furthermore, we found the microenvironment derived from metanephros transplantation was effective in inhibiting the progression of AKI induced by glycerol, which will provide a potential target or a new approach for the treatment of the acute renal injury.
